# Surface soil metal elements variability affected by environmental and soil properties

**DOI:** 10.1371/journal.pone.0254928

**Published:** 2021-07-22

**Authors:** Wei Wu, Yu Li, Mingshu Yan, Lechao Yang, Jiali Lei, Hong-Bin Liu

**Affiliations:** 1 College of Computer and Information Science, Southwest University, Chongqing, China; 2 Southeast Sichuan Geological Team of Chongqing Bureau of Geology and Mineral Exploration (Chongqing Key Laboratory of Land Quality Geological Survey), Chongqing, China; 3 College of Resources and Environment, Southwest University, Chongqing, China; CSIRO, AUSTRALIA

## Abstract

Identifying the factors controlling the spatial variability of soil metal elements could be a challenge task due to the interaction of environmental attributes and human activities. This study aimed to investigate the critical explanatory variables controlling total Ca, Cd, Cr, Cu, Zn, Fe, Mn, Mg, Pb, and Zn variations in the arable topsoil using classical statistics, principal component analysis, and random forest techniques. The work was conducted in the core region of the Three Gorges Reservoir of China. The explanatory variables included soil, topography, climate, vegetation, land use type, and distance-related parameters. Average concentrations of the metal elements were in order of Fe > Mg > Ca > Mn > Zn > Cr > Ni > Pb > Cu > Cd. Soil Cr, Fe, and Pb showed low variability while others presented medium variability. Average concentrations of Cr, Fe, Cd, and Mg exceeded their corresponding background values. There were highly positive correlations between all metal elements except Pb, Cd and Cr. The principal component analysis further demonstrated that the sources of Pb, Cd, and Cr differed with other elements. The results of random forest suggested that soil properties followed by topography were critical parameters affecting the variations of Ca, Mg, Mn, Fe, Ni, Zn, and Cu. Agricultural activities and soil properties were major factors controlling the variations of Pb, Cr, and Cd. Further study should be conducted to understand the relations between the metal elements and soil properties.

## Introduction

Soil is an important part of terrestrial ecosystems [1]. Soil metal elements, such as Co, Cu, Fe, Mn, Mo, Ni, and Zn, are essential for plant normal growth and development [2, 3]. However, some of metal elements, such as As, Cd, Hg, and Pb, that do not perform any known physiological function in plants, are not essential. In general, deficient or excessive concentrations of the metal elements may have adverse effects on plant growth, environmental quality, and human health [4, 5]. Soil metal elements mainly originate from the soil parent material [6, 7], and then are redistributed by pedological activities [5].

Topography significantly affects runoff, drainage, soil temperature, and soil erosion, which consequently results in the spatial variability of soil chemical and physical properties [8–21]. In the areas with complicated topography, landform offers a diverse geopedological condition and thus influences on the spatial distribution pattern of soil metal elements. For example, Rezapour et al. [19] analyzed the variations of total Fe, Mn, Zn, Cu, and Ni in a mountainous area. They found that the difference in the concentration of soil metal elements between topographic aspects was mainly due to the different weathering rate of source rocks on north-facing slope and south-facing slope. In the dry-hot valley of Upper Red River, the concentrations of metal elements in the topsoil were controlled by soil and topographic factors [20]. Higher concentrations of Cr, Ni, Zn, and Pb were found at lower elevation and slope areas. In a hilly area of SE Poland, the highest concentrations of total Cu and Zn occurred at the bottoms of depressions and at the foot of slopes because of soil erosion [21].

At larger scales, the effect of climate on soil metal elements variations will be enhanced. For instance, Ren et al. [22] concluded that the variations of metal elements were mainly influenced by longitude, pH, mean annual temperature and precipitation after analyzing over 50 elements based on 9830 topsoil samples across an area of 39000 km^2^ in southeast China. Martin et al. [23] reported that soil elements variations were closely linked to variations of source geology, soil type, climate and topography based on a database of the regional geochemical baseline soil survey in southern New Zealand. In southern Norway, transboundary atmospheric transport is a major source of metal elements (e.g., Pb, As, and Cd) to surface soils [24].

It has been found that anthropogenic activities resulted in relatively high concentrations of some elements (e.g., S, P, Cd, Pb, and Hg) in topsoil [25, 26]. In intensely agricultural areas, the applications of inorganic fertilizers, manures, agrochemicals, and irrigation water are the major anthropogenic sources of metal elements [25–27]. Additionally, traffic might be the main source for Pb accumulation in surface soils [28].

Identifying the critical factors influencing the variability of soil metal elements could be a challenging task due to the interaction of environmental attributes and human activities, since these factors might differ with the area of interest. For instance, some authors found that higher concentrations of soil metal elements were observed in the areas with lower elevation [20, 21], while others reported an opposite result [29]. In consideration of the potential adverse effects of metal elements on plant growth, the current study made an attempt to investigate the variations of total metal concentrations (Ca, Cd, Cr, Cu, Zn, Fe, Mn, Pb, Mg, and Ni) in the cultivated soils within an intensely agricultural area. The objectives were to (1) analyze the concentrations and variations of soil metal elements and (2) determine relative importance of explanatory variables (e.g., soil, topography, climate, vegetation, land use type, and distance-related parameters) controlling metal elements variations. Specifically, two well-known techniques, i.e. principal component analysis (PCA) and random forest (RF), were used in this work.

## Materials

### Study area

**Fig 1:**
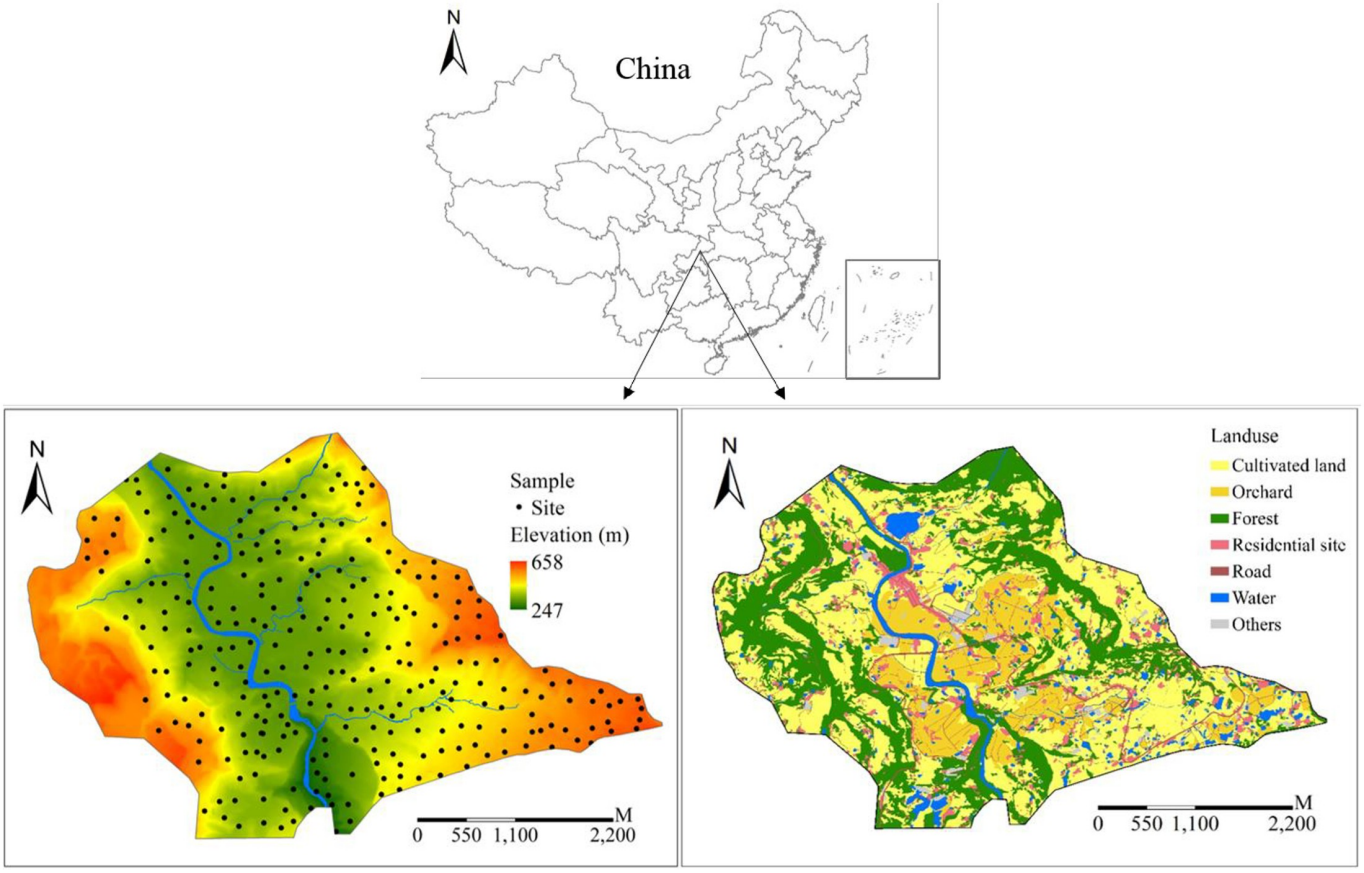
Maps of study area locations, soil sample sites, digital elevation model, and land use type.

The study area (108°13’-108°18’E, 30°39’-30°42’N) covering about 18 km^2^ is located in the Ganning town in the core region of the Three Gorges Reservoir of China (Fig 1). The elevation varies between 247 and 658 m with a mean of 433 m. The slope changes between 0.45° and 63.9° with a mean of 12.72°. It has a humid subtropical monsoon climate characterized by hot summer and warm winter. Annual precipitation is 1293 mm which mainly occurs in summer. Mean annual temperature is 17°C. Average annual sunshine hour is 1204.5 h and relative humidity is 81%. The frost free period varies between 260 and 283 d. The Ganning river with five streams flows through the study site. This resulted in the flat valley bottom lying in the middle of the area. The main land use types are paddy field (23.63%), dry land (16.89%) and orchard (12.48%). The dry land is mostly located in the areas with elevation (mean = 432 m) varying between 252 and 650 m and slope (mean = 11.8°) changing between 0 and 55°. The paddy field is sited in the areas with elevation (mean = 461 m) varying between 265 and 651 m and slope (mean = 7°) changing between 0 and 25°. The orchard is mainly distributed in lower places with elevation (mean = 382 m) varying between 269 and 617 m and slope (mean = 7°) changing between 0 and 26°. The dryland is planted with winter rapeseed (*Brassica napus* L.)—corn (*Zea mays* L.) or sweet potato (*Ipomoea batatas* L.). The orchard is grown with blood oranges (*Citrus sinensis* (L.) Osbeck). The paddy field is planted with single rice (*Oryza sativa* L.) from April to September and retains water in winter. In the current study area, farmers generally tend to apply inorganic fertilizers, manures, and urea. They seldom applied potassium fertilizer in agricultural activities.

The soil parent material is Shaximiao formation which was deposited during the middle Jurassic period. Soil developed from this geological unit is classified as pup-orthic-Entisol, Regosols, and Entisol according to the Chinese Soil Taxonomy, the FAO Soil Classification, and the USDA taxonomy, respectively [30]. The parent material is Shaximiao formation which was deposited during the middle Jurassic [30]. Soil pH varies between 4.63 and 8.43 with a mean of 6.23.

### Soil sampling and analysis

Within the study area, 300 samples (paddy field: 127, dryland: 98, orchard: 75) were collected based on a mixture of random and systematic sampling strategies. The study did not involve private land, protected land, endangered or protected species. No specific permissions were required for these locations/activities. Fig 1 showed the distribution map of the sampling sites. The points were selected to cover the entire study area. The work was conducted in March, 2019. For each sampling site, a mixed sample (3 to 5 subsamples within 5–10 m radius of the site) with an approximate weight of 500 g were collected. All the sampling locations were recorded by Global Positioning System (GPS). Standard measurements were performed on the soil samples. Samples were air-dried and passed through a 2 mm soil sieve before laboratory analyses.

Soil chemical analyses were conducted according to the methods recommended by CGS [31, 32]. Soil pH was measured in 1:2.5 soil-water suspension with a pH meter. Soil organic matter (SOM) was determined using the oil bath-K_2_Cr_2_O_7_ titration method [33] and total nitrogen (N) was measured using the Kjeldhal method [34]. Soil organic carbon (SOC) was calculated based on the assumption that SOM contains 58% carbon. Total calcium (Ca), chromium (Cr), iron (Fe), manganese (Mn), phosphorus (P), and potassium (K) were determined by X-Ray Fluorescence Spectrometry (XRFS) method [31, 32]. Total cadmium (Cd), copper (Cu), nickel (Ni), lead (Pb), and zinc (Zn) were measured by Inductively Coupled Plasma-Mass Spectrometry (ICP-MS) method [31, 32]. Total magnesium (Mg) was quantified by Inductively Coupled Plasma-Optical Emission Spectrometry (ICP-OES) method. The accuracies and precisions of all elements were satisfied with the analytical requirements of CGS [31, 32].

### Explanatory variables

A set of climate, topography, vegetation, land use type, soil property (pH, SOC, N, K, and P), and distance-related parameters were used to explain the variability of soil metal elements (Table 1 and Fig 1). The topographic factors were elevation, slope, and aspect, which were calculated from a digital elevation map (DEM) with a resolution of 5 m using SAGA GIS software [35]. The climate parameters including long-term mean temperature (MAT) and precipitation (MAP) were obtained from the WorldClim Database (http://www.worldclim.org) with a resolution of 1000 m [36]. The long-term normalized difference vegetation index (NDVI) with a time resolution of 16 days and a spatial resolution of 250 m was downloaded from LAADS DAAC (http://ladsweb.nascom.nasa.gov), and the averaged NDVI as a proxy for vegetation was calculated with ArcGIS v.10.5 software. Three land use types were dryland, paddy field, and orchard, which were recorded during soil sampling. Distance to river (D2River), house (D2House), and road (D2Road) was calculated for each site.

**Table 1:** Explanatory variables used in the study.

Attribute	Brief description	Unit
**Elevation**	**Land surface elevation above mean sea level**	**m**
**Slope**	**Maximum rate of change between cells and neighbors**	**Degree**
**Aspect**	**Direction of the steepest slope from the north**	**Degree**
**MAT**	**Long-term mean temperature**	**°C**
**MAP**	**Long-term mean precipitation**	**mm**
**NDVI**	**Normalized difference vegetation index**	**Non-dimensional**
**Land use type**	**Three land use types: orchard, dry land, and paddy field**	**Non-dimensional**
**D2Road**	**Distance to road**	**m**
**D2House**	**Distance to house**	**m**
**D2River**	**Distance to river**	**m**
**pH**	**Soil pH**	
**N**	**Soil total nitrogen**	**mg/kg**
**P**	**Soil total phosphorus**	**mg/kg**
**K**	**Soil total potassium**	**%**
**SOC**	**Soil organic carbon**	**%**

### Methodology

#### Statistical analysis

Spearman correlation analyses were applied to calculate the relationships between soil metal elements and explanatory variables. Mann-Whitney test was used to evaluate the difference in soil metal elements between land use type.

#### Principal component analysis (PCA)

Principal component analysis (PCA) is a well-known method for dimension reduction. In PCA, the *d*-dimensional data are reduced into a lower-dimensional space by creating a set of new orthogonal variables (principal components) based on the original dataset. Variables input for PCA included the studied metal elements (Ca, Cr, Cu, Fe, Mn, Pb, Zn, Cd, Mg, and Ni). The data were examined by Kaiser-Meyer Olkin (KMO) and Bartlett tests. The KMO test is used for evaluating the number of samples and the Bartlett test for homogeneity of variances. The PCA result was rotated using varimax with Kaiser normalization method to minimize the variations among the variables for each factor. A principal component with an eigenvalue > = 1 was considered. These principal components were then used as the dependent variables for random forest to explore the effects of environmental and soil properties on metal elements.

#### Random forest

Random forest (RF) was proposed by Breiman in 2001 [37]. It creates a forest based on bagging [37] and random features selection [38–40]. Bagging generates a set of subsets of the original dataset uniformly and with replacement. Each individual tree is trained on the bootstrapped samples from the training data and tested on the reminders. During this procedure, a random subset of features is selected to split the nodes of the tree. Then, diverse but approximately unbiased base models are created and this could guarantee the predictive accuracy of random forest. It contains a set of classification and regression trees. RF implements a classification task if the response is a categorical variable and carries out a regression job if the response is a continuous variable. For classification tasks, the results are based on a majority voting strategy. For regression tasks, the predictions are the averages of the trees according to the following equation,

$$\overset{-}{t}(x)=\frac{1}{n}\sum_{i=1}^{n}t(X;v_{i})$$ (1)

where $\overset{-}{t}(x)$ is a predicted value, *n* is the number of trees, *t(X; v*_*i*_*)* is an outcome of a tree, *X* is an input matrix, *v*_*i*_ is the *i*th random vector having independent and uniformly distributed.

Each tree is trained by about two-third of the data and tested by the others. The testing data are out-of-bag (OOB) samples and the OOB error is calculated [37]. The OOB error is an unbiased estimator. It is similar with the prediction error produced by an independent testing dataset. The mean square error (MSE_OOB_) of a forest is calculated by

MSEOOB=1n∑i=1n(ti−t^iOOB)2 (2)

where t_i_ is the OOB prediction of the ith sample, ${\hat{\text{t}}}_{{\text{i}}_{\text{OOB}}}$ is the average of OOB predictions.

Additionally, variable importance is calculated based on mean decrease in accuracy (MDA) and mean decrease in Gini (MGD) [37]. The MDA measures a variable importance according to its contribution to the prediction accuracy. The MDG measures a variable importance according to the split quality of a decision tree based on the variable. A variable with higher homogeneity in splitting has a higher MGD [37]. In the current work, the variable importance was produced by MDA, since it is more reliable than MDG [37]. The relative importance of each explanatory variable was then calculated based on MDA.

For RF, the obtained principal components were response variables, the environmental (topography, climate, vegetation, land use, distance-related parameters) and soil properties (N, P, K, SOC, pH) were explanatory variables.

#### Performance evaluation

Leave one out cross validation method was applied to assess model performance based on a set of statistical error indicators. Coefficient of determination (R^2^), root mean square error (RMSE), and Lin’s concordance correlation coefficient (LCCC) were used in the current study.

$${\text{R}}^{2}=\frac{\sum_{i=1}^{n}{\left(Q_{pi}-{\overset{-}{Q}}_{o}\right)}^{2}}{\sum_{i=1}^{n}{\left(Q_{oi}-{\overset{-}{Q}}_{o}\right)}^{2}}$$ (3)

$$\text{RMSE}=\sqrt{\frac{\sum_{i=1}^{n}{\left(Q_{oi}-Q_{pi}\right)}^{2}}{n}}$$ (4)

$$\text{LCCC}=\frac{2R\sigma_{o}\sigma_{p}}{\sigma_{o}^{2}+\sigma_{p}^{2}+{\left({\overset{-}{Q}}_{o}+{\overset{-}{Q}}_{p}\right)}^{2}}$$ (5)

where *n* is the number of data, *Q*_*oi*_ and *Q*_*pi*_ are the measured and predicted contents of the *i*th soil sample, respectively, $\overset{-}{Q_{o}}$ and $\overset{-}{Q_{p}}$ are the mean values of the measurement and prediction, σ_o_ and σ_p_ are the variances of the measurements and predictions. The most accurate model has the lowest value of RMSE and highest values of R^2^ and LCCC. The LCCC measures the agreement between measured and predicted values. It combines both precision and bias to determine how far the data deviate from the 1:1 line. The value of LCCC changes between -1 and 1, with 1 representing perfect agreement, 0.9–1 excellent agreement, 0.8–0.9 substantial agreement, 0.65–0.8 moderate agreement, and values <0.65 poor agreement [41].

#### Software

Basic statistical analyses and PCA were performed with SPSS v18.0. Model development and evaluation were conducted by using R v3.6.1 (http://www.r-project.org). The package of randomForest implemented in R software was used in the current study. A combination of ntree {(100, 3000), 100} and mtry {1, 15} was tested to optimize the parameters for RF. Finally, the optimal parameters of ntree and mtry were 2900, 5 for PCA1 and 200, 9 for PCA2, respectively. All the calculations were performed in a computer with Intel Core CPU i3-4160 @ 3.6 GHz and 4 GB of RAM memory.

## Results

### Preliminary analysis

The basic statistics of the metal concentrations and explanatory parameters were shown in Table 2, with last column presenting the background values for the same metal elements in China [42]. Average concentrations of the metal elements were in the following descending order: Fe > Mg > Ca > Mn > Zn > Cr > Ni > Pb > Cu > Cd. The 300 soil samples showed average values of Ca, Cu, Mn, Pb, Zn, and Ni below their corresponding background values, while others slightly surpassed the values. Three soil samples had higher concentration of Ca than the background value. About half of the samples presented higher concentrations of Cu (49%), Mn (45%), Pb (43%), Zn (46%), and Ni (54%) than their background values. About 70% samples had higher concentrations of Cr (71%), Fe (71%), and Mg (76%) than their background values. Almost all samples (98%) had higher concentration of Cd than its background value, indicating that the analyzed sampling sites within the study area were notably accumulated Cd. In terms of coefficient of variation (CV%), Cr, Fe, and Pb showed low variability (<25%) while others presented medium variability (25%-75%). For the explanatory parameters, elevation, MAT, MAP, NDVI, soil pH, and K had low variability, slope, D2Road, and D2River displayed high variability, and others showed medium variability.

**Table 2:** Summary statistics of concentrations of metal elements and explanatory parameters (sample number = 300).

	Min	Max	Mean	Stdev	CV (%)	BK
**Ca (mg/kg)**	**1635**	**20819**	**7478**	**2895.6**	**38.72**	**15400**
**Cr (mg/kg)**	**36.4**	**95.68**	**64.39**	**10.82**	**16.81**	**61**
**Cu (mg/kg)**	**7.441**	**36.634**	**20.82**	**5.88**	**28.25**	**22.6**
**Fe (mg/kg)**	**15301**	**45580**	**32320**	**6650.9**	**20.58**	**29400**
**Mn (mg/kg)**	**129.7**	**1059.5**	**527**	**198**	**37.61**	**583**
**Pb (mg/kg)**	**17.7**	**34.7**	**25.53**	**2.31**	**9.04**	**26**
**Zn (mg/kg)**	**21.94**	**145.42**	**68.22**	**18.21**	**26.70**	**74.2**
**Cd (mg/kg)**	**0.070**	**0.540**	**0.221**	**0.07**	**31.31**	**0.097**
**Mg (mg/kg)**	**2912**	**17649**	**11237**	**4167**	**37.08**	**7800**
**Ni (mg/kg)**	**9.31**	**50.1**	**25.79**	**7.63**	**29.58**	**26.9**
**Elevation (m)**	**252.28**	**625.00**	**420.23**	**93.53**	**22.26**	
**Slope (°)**	**0**	**54.87**	**8.64**	**7.66**	**88.58**	
**Aspect (°)**	**0**	**360**	**207**	**113**	**54.67**	
**MAT (°C)**	**16.46**	**17.54**	**17.13**	**0.29**	**1.70**	
**MAP (mm)**	**1277**	**1289**	**1282**	**3.39**	**0.26**	
**NDVI**	**0.50**	**0.64**	**0.58**	**0.03**	**4.63**	
**D2Road (m)**	**0.07**	**333**	**65.91**	**51.01**	**77.39**	
**D2House (m)**	**0.12**	**189.9**	**62.28**	**35.8**	**57.48**	
**D2River (m)**	**1.16**	**1255**	**353.46**	**285.33**	**80.73**	
**pH**	**4.63**	**8.43**	**6.26**	**0.83**	**13.22**	
**N (mg/kg)**	**381**	**1660**	**888.3**	**243.6**	**27.43**	
**P (mg/kg)**	**196.35**	**2474.9**	**627.64**	**253.08**	**40.32**	
**K (%)**	**0.998**	**3.028**	**2.064**	**0.42**	**20.31**	
**SOC (%)**	**0.147**	**1.404**	**0.787**	**0.209**	**26.53**	

CV: coefficient of variation; BK: background values [42].

Table 3 showed the Spearman correlation coefficients between metal elements. Of course, most of them were closely correlated. Lower correlation coefficients (< 0.6) existed between Pb, Cd and Cr with most metal elements. Others presenting higher coefficients suggested common origin of these metal elements in the study area.

**Table 3:** Spearman coefficients between metal elements (sample number = 300, p<0.05).

	**Ca**	**Cu**	**Fe**	**Mn**	**Zn**	**Mg**	**Ni**	**Cr**	**Cd**
**Cu**	**0.694**								
**Fe**	**0.698**	**0.892**							
**Mn**	**0.74**	**0.678**	**0.728**						
**Zn**	**0.76**	**0.898**	**0.874**	**0.682**					
**Mg**	**0.846**	**0.847**	**0.886**	**0.746**	**0.875**				
**Ni**	**0.702**	**0.878**	**0.904**	**0.687**	**0.85**	**0.898**			
**Cr**	**0.506**	**0.781**	**0.835**	**0.541**	**0.731**	**0.741**	**0.866**		
**Cd**	**0.456**	**0.516**	**0.458**	**0.369**	**0.634**	**0.495**	**0.523**	**0.45**	
**Pb**	**0.197**	**0.59**	**0.582**	**0.286**	**0.56**	**0.402**	**0.579**	**0.592**	**0.5**

**Fig 2:**
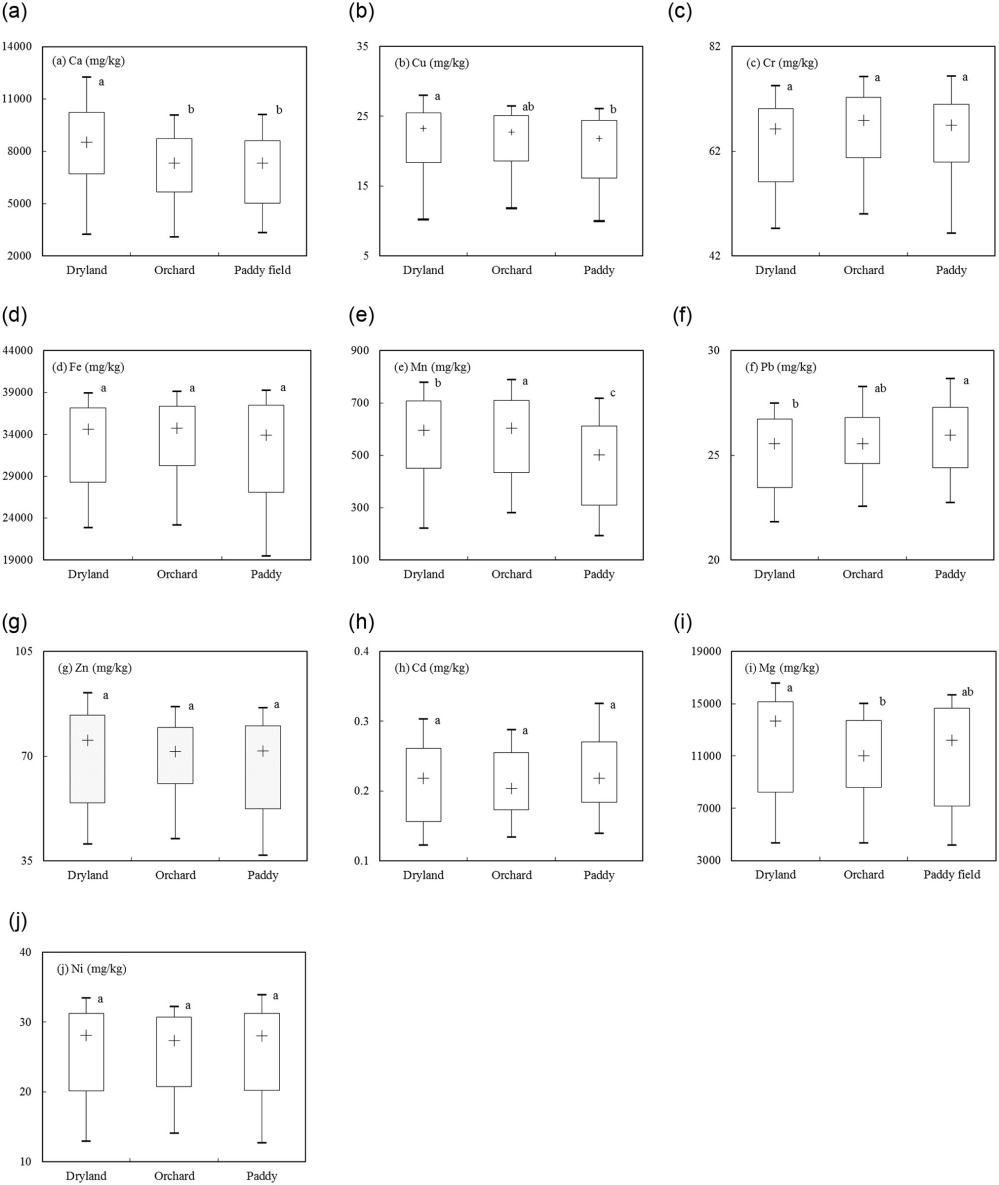
Box plots (in 10-25-50-75-90th percentiles) of metal elements under dryland, orchard and paddy land use (different letters within each plot indicate significant difference in metal element between the land use type at p<0.05).

Results of Mann-Whitney U-test indicated that significant differences in Ca, Cu, Mn, Pb, and Mg concentrations of existed between land use types (Fig 2). The orchard had significantly higher concentrations of Mn and lower concentrations of Ca and Mg. The dryland had significantly higher concentrations of Ca, Cu, Mg and lower concentrations of Pb. The paddy fields had significantly lower concentrations of Ca, Cu, Mn and higher concentrations of Pb.

Table 4 showed Spearman correlation coefficients between metal elements and explanatory variables. Some of them were closely correlated. Specifically, all metal elements were highly correlated with P, K, and topographical aspect (p < 0.05). Nine of them (except Pb) were closely correlated with elevation and pH.

**Table 4:** Spearman coefficient between metal elements and explanatory variables (sample number = 300, p<0.05, NS indicates no significance).

	Ca	Cr	Cu	Fe	Mn	Pb	Zn	Cd	Mg	Ni
**Ele**	**-0.254**	**-0.192**	**-0.261**	**-0.245**	**-0.295**	**NS**	**-0.205**	**-0.12**	**-0.202**	**-0.199**
**Slope**	**0.223**	**NS**	**NS**	**NS**	**0.165**	**-0.141**	**0.151**	**NS**	**0.211**	**NS**
**Aspect**	**-0.217**	**-0.24**	**-0.252**	**-0.158**	**-0.228**	**-0.198**	**-0.231**	**-0.169**	**-0.191**	**-0.213**
**Temp**	**NS**	**NS**	**NS**	**NS**	**NS**	**NS**	**NS**	**NS**	**NS**	**NS**
**Rain**	**NS**	**NS**	**0.162**	**0.13**	**0.129**	**0.163**	**0.124**	**NS**	**0.12**	**NS**
**NDVI**	**NS**	**NS**	**NS**	**NS**	**NS**	**-0.147**	**NS**	**NS**	**NS**	**NS**
**pH**	**0.637**	**0.327**	**0.345**	**0.373**	**0.39**	**NS**	**0.394**	**0.353**	**0.484**	**0.46**
**SOC**	**-0.266**	**NS**	**NS**	**NS**	**-0.384**	**0.309**	**NS**	**0.18**	**-0.207**	**NS**
**N**	**-0.139**	**0.193**	**0.127**	**NS**	**-0.267**	**0.483**	**0.194**	**0.389**	**NS**	**NS**
**P**	**0.569**	**0.286**	**0.512**	**0.419**	**0.447**	**0.168**	**0.58**	**0.311**	**0.513**	**0.381**
**K**	**0.73**	**0.709**	**0.809**	**0.812**	**0.672**	**0.449**	**0.845**	**0.489**	**0.898**	**0.86**
**D2Road**	**NS**	**NS**	**NS**	**NS**	**NS**	**NS**	**NS**	**NS**	**NS**	**NS**
**D2River**	**-0.157**	**-0.136**	**-0.137**	**-0.128**	**NS**	**NS**	**NS**	**-0.13**	**-0.126**	**-0.117**
**D2House**	**-0.209**	**NS**	**-0.195**	**-0.151**	**-0.162**	**-0.133**	**-0.188**	**NS**	**-0.127**	**NS**

### Principal component analysis

The value of KMO value was 0.911 and significant value of Bartlett was less than 0.001 indicating that the PCA result was acceptable. Table 5 showed the results of PCA analysis. The proportion of variance explained by the first two principal components (PCA) was approximately 83.2%. The variables with the highest loadings in PCA1, which accounted for about 48.1% of the total variance, were Ca > Mg > Mn > Fe > Ni > Zn > Cu. All these metals were probably related to lithology. In PCA2, the metals Pb > Cr > Cd had the highest loading values and explained 35.1% of the total variance. These metals were probably affected by both lithology and agronomic practices, such as fertilization. In this case, Pb and Cd were positively correlated with SOC while Ca, Mn, and Mg were negatively correlated with SOC (p < 0.05), also suggesting that the origin of these metals might be different.

**Table 5:** Summary of principal component analysis (PCA).

	PCA1	PCA2
**Ca**	**0.909**	**0.091**
**Cr**	**0.544**	**0.722**
**Cu**	**0.709**	**0.625**
**Fe**	**0.753**	**0.596**
**Mn**	**0.857**	**0.223**
**Pb**	**0.086**	**0.919**
**Zn**	**0.734**	**0.587**
**Cd**	**0.258**	**0.646**
**Mg**	**0.864**	**0.441**
**Ni**	**0.723**	**0.619**
**Proportion of variance%**	**48.1**	**35.1**
**Cumulative proportion%**	**48.1**	**83.2**

Extraction method: Principal component analysis

Rotation method: Varimax with Kaiser normalization

### Model performance and variable importance

Random forest was used to explore the effects of the explanatory variables on the two principal components (PCA1 and PCA2) over the study area. The accuracy indicators of out of bag (OOB) and leave one out cross validation (CV) were given in Table 6. The errors of OOB and CV for both PCA1 and PCA2 gave similar values suggesting the stability of RF. About 80% and 53% variations of PCA1 and PCA2 could be explained by the explanatory variables, respectively. In terms of LCCC, models gave substantial agreement for PCA1 and moderate agreement for PCA2.

**Table 6:** Coefficient of determination (R^2^), root mean square error (RMSE) and Lin’s concordance correlation coefficient (LCCC) of out of bag (OOB) and leave one out cross validation (CV) of random forest for the first two principal components (PCA1 and PCA2).

	R^2^_OOB_	RMSE_OOB_	LCCC_OOB_	R^2^cv	RMSEcv	LCCCcv
**PCA1**	**0.8**	**0.447**	**0.875**	**0.817**	**0.447**	**0.875**
**PCA2**	**0.529**	**0.685**	**0.682**	**0.532**	**0.684**	**0.682**

**Fig 3:**
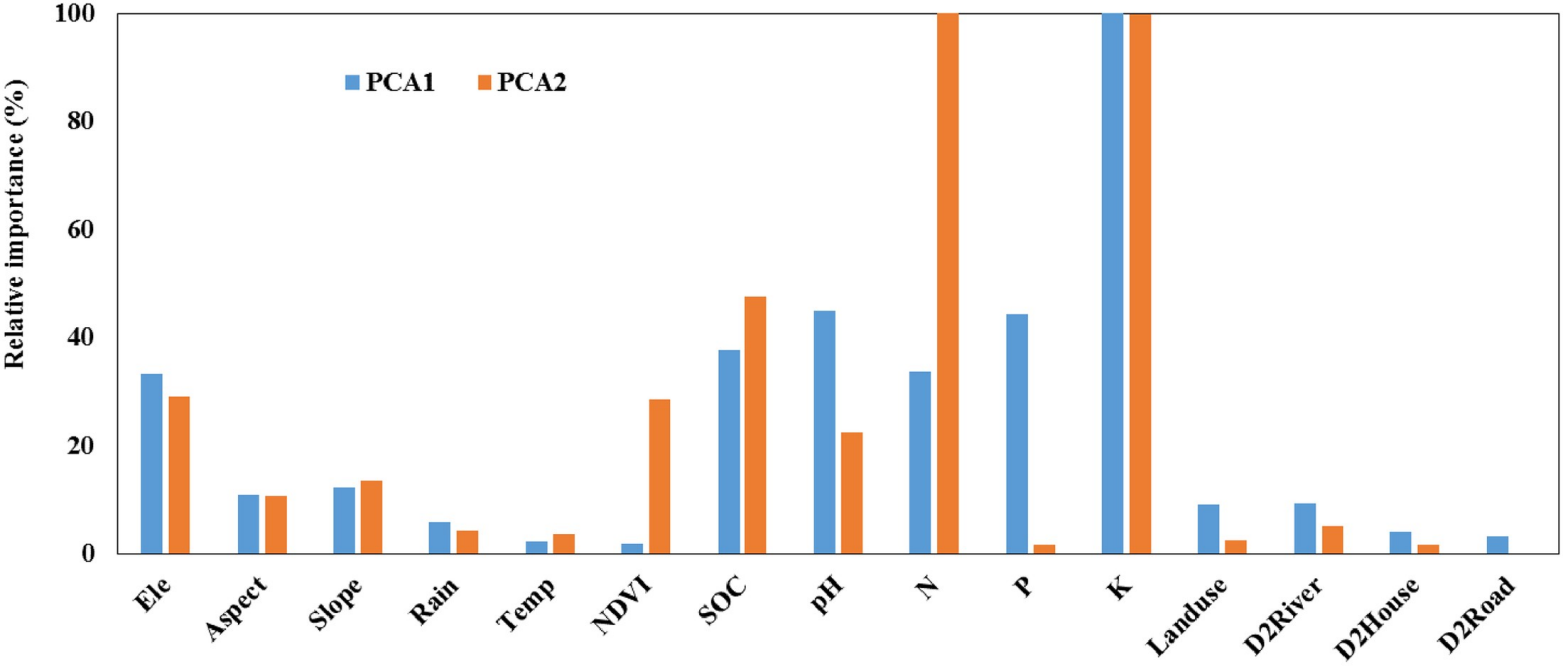
Variable relative importance to PCA1 and PCA2 produced by RF models.

Fig 3 illustrated the variable relative importance on PCA1 and PCA2. Obviously, soil properties followed by terrain indicators presented higher values of relative importance for both PCA1 and PCA2. For PCA1, the most important variable was K which had much higher relative importance value than others. For PCA2, N and K had much higher relative importance values. Topographical factors showed similar relative importance to PCA1 and PCA2. Climate and distance-related parameters had very low relative importance values. Soil pH and P showed relative higher values for PCA1 than for PCA2. NDVI had much higher values for PCA2 than for PCA1.

## Discussion

The ten metal elements were reduced to two principal components (PCA1 and PCA2) according to their homogeneity (Table 5). The higher effects of K on both PCA1 and PCA2 indicated that these metal elements were mainly controlled by the lithogenesis over the study area, since the farmers seldom applied K-fertilizers in agricultural activities. Besides, some authors have reported that soil Ni is highly associated with the processes of weathering and pedogenesis [43, 44]. This further indicated that the variations of the metal elements in PCA1 were strongly linked to soil development process over the current study area. The effect of N on PCA2 revealed by random forest suggested that the metal elements (Cr, Pb, and Cd) in PCA2 were also closely related with N-fertilizer over the study area, because the farmers are used to apply large amounts of N-fertilizers to get more yields. Additionally, in intensely agricultural areas, the accumulation of Cd was often attributed to fertilizer applications [25]. Soil pH showed higher importance to PCA1 due to the strong relationship between pH and Ca (r = 0.64, p < 0.05, Table 4), since Ca was involved in PCA1. Meanwhile, the lower relationships between soil P and the metal elements in PCA2 could explain the lower importance of P to PCA2. However, the relationship between K and the metal elements are unclear and further studies are needed.

Topography plays an important role in soil formation and hence might affect soil metal elements variability. In the current study, topography was the second important parameter affecting the metal elements variability. Elevation followed by slope showed highest importance to both PCA1 and PCA2 (Fig 3). According to the Spearman correlation analysis, higher concentrations of metal elements (except Pb) existed in the areas with lower elevation. This was in consistent with the reported findings that fine particle-related metals (e.g., Cr, Cu, Zn, Ni, and Cd) are prone to accumulate at low-elevation areas [45]. Besides, in the current study site, the parent material is composed of purple sandstone and purple shale rock and is sensitive to physical weathering, such as gravitational collapse [46]. Therefore, metal elements were prone to move to the lower areas with the fine fraction of the soil and thus higher concentrations of these elements mostly existed in these areas.

NDVI which reflects the vegetation growth status had much higher importance to PCA2 than to PCA1 (Fig 3). Soil Pb involved in PCA2 was the only metal that was significantly negatively correlated with NDVI. These suggested that the plants growing in the study area might be injured by Pb.

Although the distance to river and to house had very low relative importance to metal elements variations, the Spearman correlation coefficients showed that there were negative relationships between the distance-related indices and some metal elements. This indicated that human daily activities, such as washing, might result in these metal accumulation in the area around the houses and thus along the river. It has been reported that Pb in surface soils was often accumulated by traffic [28]. However, the relative importance of the distance to road and the Spearman correlation coefficients indicated that variation of Pb was probably ascribed to agricultural practices over the current study area.

## Conclusions

The current study applied classical statistics, principal component analysis, and random forest to investigate the critical explanatory variables controlling the variability of metal elements in the arable topsoil. The main findings are as follows,

1. The soil metal elements showed low to medium variability. The Spearman coefficients between the metal elements varied from 0.197 to 0.904 (p < 0.05). The first two principal components explained about 83.2% of the total variance.
2. Soil properties followed by topography are critical parameters affecting the variations of Ca, Mg, Mn, Fe, Ni, Zn, and Cu. Agricultural activities and soil properties are major factors controlling the variations of Pb, Cr, and Cd.

However, the effects of explanatory variables on metal elements are complex. Each variable could exert its influence in different ways simultaneously. Besides, conjoining influences may restrain or nullify each other. For instance, the effects of climate parameters and land use type could be eliminated or replaced by topography. Some terrain indicators, such as elevation and aspect, are closely related with the local micro-climate conditions. The distribution of land use type is usually determined by topography, for example, the orchard and dryland located in lower areas and paddy field in higher places in this case.

## Supporting information

S1 File Data. (CSV)
